# The work-relatedness at a case of acute lymphoblastic leukemia in a radiation oncologist

**DOI:** 10.1186/s40557-017-0186-8

**Published:** 2017-06-27

**Authors:** Bong Hyun Kim, Young-Jun Kwon, Young-Su Ju, Bong Kyu Kim, Hyun Seok Lee, Sang-gil Lee, Yun Kyung Chung

**Affiliations:** 1 Department of Occupational and Environmental Medicine, Hallym University Sacred Heart Hospital, Dongangu 772, Anyang city, Republic of Korea; 2 Occupational Safety and Health Research Institute, Korea Occupational Safety and Health Agency, Ulsan city, Republic of Korea

**Keywords:** Acute lymphoblastic leukemia, Radiation oncologist, Cesium, Brachytherapy

## Abstract

**Background:**

Clinicians who perform radiation therapy (RT) are exposed to radiation, which may negatively affect their health. The present study reports a case of acute lymphoblastic leukemia in a healthcare provider who was exposed to radiation at work; we also present a literature review of this topic.

**Case presentation:**

A 45-year-old patient, who had been a radiation oncologist and had been exposed to radiation while performing brachytherapy 10 years ago, complained of chest pain and was suspected of having leukemia based on the results of a blood test in an outpatient clinic. He was diagnosed with acute lymphoblastic leukemia, and subsequently underwent chemotherapy. However, the case died during treatment. Through epidemiological investigation, it was found that the case’s cumulative exposure dose based on personal exposure and spatial dose measured during the work period was in the range of 6.08–12.15 mSv.

**Conclusions:**

Based on the following considerations, acute lymphoblastic leukemia was highly correlated with the level of radiation to which the case was exposed while performing brachytherapy on patients with cancer. Firstly, the latent period of acute lymphoblastic leukemia in the case closely matched the latency time reported in previous published studies (5–10 years). In addition, numerous studies have reported significantly higher relative risks of cancer among clinicians who perform RT compared with the general population. The case was also atypically exposed to radiation through his hands, despite wearing protective equipment. Lastly, the case’s coworkers were also found to have been exposed to high levels of radiation. Investigation into the influence of radiation exposure through atypical routes during RT on the health of clinicians is recommended.

## Background

In the United States, the number of radiologic interventions increased by 7,000,000 cases per year between 1980 and 1982, reaching 18,000,000 per year by 2006 [1].

The number of workers engaged in radiation work in Korea was 12,625 in 1995, and dramatically increased to 76,493 in 2015. The mean dose of personal radiation exposure among them, as measured with personal radiation dosimeters carried by each worker, was 0.39 mSv per year in 2015. This measurement has been consistently decreasing from 0.95 mSv in 2004, the year in which the statistical analysis was initiated, to 0.77 mSv in 2006, 0.67 mSv in 2008, 0.58 mSv in 2010, 0.48 mSv in 2012, and 0.41 mSv in 2014. Considering the mean exposure dose per year by occupation, radiologists had the highest mean exposure dose of 0.8 mSv, followed by miscellaneous occupations with 0.31 mSv, and doctors with 0.3 mSv. The number of workers engaged in radiation work whose radiation exposure dose exceeded 5 mSv/quarter was 569, accounting for 0.7% overall. Of 16,330 doctors, 170 (1.0%) had a mean radiation exposure dose greater than 5 mSv/quarter, and 10 doctors (0.1%) had a mean radiation exposure dose greater than 20 mSv/quarter [2]. Although radiologic interventions have conferred many medical benefits for patients with cancer, they can negatively affect the health of healthcare providers who routinely perform radiation therapy (RT).

Radiation exposure above the threshold dose has detrimental effects such as cataracts, skin fibrosis, alopecia, and infertility. It also has probabilistic or stochastic effects, which are not associated with the threshold dose but more with genetic effects, and may induce leukemia, breast cancer, and ovarian cancer [3].

Acute lymphoblastic leukemia is a malignant condition in which lymphoid cell precursors proliferate excessively within the bone marrow in an immature state, thus inhibiting normal hematogenous functions. Philadelphia chromosome-positive acute lymphoblastic leukemia differs significantly from other types of acute lymphoblastic leukemia both clinically and physiologically, and accounts for 20%–30% of all cases [4]. Radiation oncology is a medical specialty focusing on RT for patients with cancer. Clinicians are exposed briefly to radiation during the process of injecting a radioactive substance into a Gamma Knife or other device. In certain treatments such as brachytherapy, clinicians are routinely exposed to relatively high levels of radiation during the process of manually injecting radioactive substances, applying dressings, or performing visual inspection. The procedures performed in the case have since been automated and no longer require manual.

We report a case of acute lymphoblastic leukemia in a former radiologic oncologist with a history of occupational radiation exposure 10 years previously, and present a literature review of acute lymphoblastic leukemia.

## Case presentation

### Patient information

Male, 45 years old

### Chief complaint

Chest pain and myalgia

### Smoking status and alcohol use

Not applicable

### History of present illness

A 45-year-old male patient, who was a former radiologic oncologist, presented to the department of rehabilitation medicine at a university hospital with chief complaints of chest pain and myalgia on August 19, 2011. The case was suspected of having a hematological malignancy based on his blood test results, and subsequently underwent a bone marrow test on August 23, 2011. On August 30, 2011, The case was diagnosed with leukemia based on histological findings. The case received chemotherapy thereafter, but died during a chemotherapy session on January 6, 2012.

### Past medical history

The case underwent surgery for cryptorchidism in 1990 and for L4/5 disc herniation in 2007. He also underwent treatment for acute gastric ulcers in 2004. In November 2000, while he was performing brachytherapy, The case experienced extreme fatigue, dramatic weight loss, and loss of appetite. In early December of the same year, he underwent extracorporeal shock wave lithotripsy after finding urinary calculi, but did not undergo any test related to excessive radiation exposure. The case started taking medicine for dyslipidemia, especially high triglyceride (TG = 473 g/dL) detected during a health examination in July 2011. No abnormal hematological findings were noted in the general health examinations recorded since 2003. The case did not have a history of any other disorders or a family history of leukemia.

### Laboratory findings

A blood test was performed in August 2011 in response to the chief complaints mentioned previously. The test showed that the patient’s level of hemoglobin was 11.1 g, white blood cells were 41,490 μl, hematocrit was 31.8%, and platelets were 49,000 μl. Based on these results, The case was suspected of having acute lymphoblastic leukemia, and accordingly, he underwent a bone marrow biopsy. Based on the bone marrow test results, The case was diagnosed with B-precursor lymphoblastic leukemia with a *BCR/ABL* rearrangement. The FISH (Fluorescence in situ hybridization) test result was *BRC/ABL1* positive, *PML/RARA* negative, and showed an *ICH* rearrangement.

### Occupational history

The case had been transferred to work as a visiting radiologic oncology researcher at the A National Hospital specializing in cancer screening and treatment on June 1, 2000. The case’s tasks included writing patient medical records, analyzing radiation doses, and observing cancer treatment. His main task was performing RT. The case was responsible for all procedures in RT including the injection/removal of radioactive isotopes and application of a dressing on the injection site within 72 h after injection. In August 2000, a nationwide doctors’ strike related to the separation of prescribing and dispensing drugs paralyzed medical institutions and their healthcare services. As a result, a very large number of patients presented to the A National Hospital where The case worked at that time, significantly increasing the patient’s workload. The number of patients treated during this period can be estimated from the number of patients who were discharged during the same period. Approximately six patients were admitted to the hospital and were discharged after undergoing brachytherapy every week. During the 6 months between August 2000 and February 2001, the case performed brachytherapy on approximately 150 patients with cancer. The case retired from his job at the A National Hospital on February 28, 2001, and started working again at the B university hospital between March 1, 2001 and February 28, 2002. He then worked as a medical school professor at the university hospital from March 1, 2002 until his death due to lymphoblastic leukemia. During this period, The case did not handle radioactive isotopes himself as he used to in the A National Hospital.

### Occupational task

**Fig. 1:**
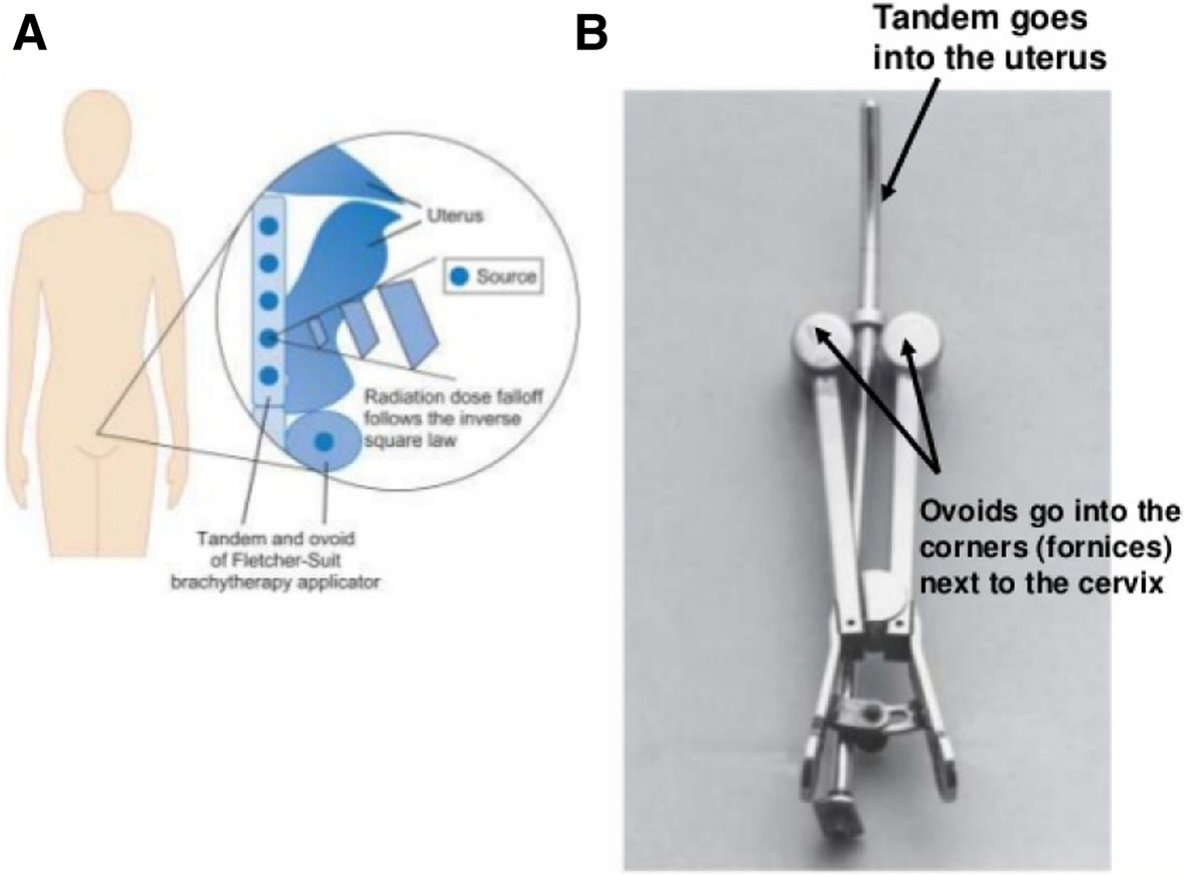
**a** Location of the radioactive source, **b** Treatment tools used in brachytherapy of patients with cervical cancer. Cervix Cancer and Radiation, Available at https://youtu.be/20s1Gq4t7nY. Accessed 22 Feb 2016.

The case mainly performed brachytherapy on patients with cervical cancer. A radioactive source such as the one shown in Fig. 1 would be inserted into the cervix of these patients, and after making sure that the radioactive source was correctly positioned, radioactive isotopes were manually injected into a designated space within the radioactive source. As shown in Fig. 1, three radioactive isotopes were placed in the middle tandem, and one in the ovoid on both sides (Fig. 1). The radioactive isotope used was cesium. Although the case’s coworkers claim that all medical specialists wore protective equipment such as lead shielding aprons and neck collars while performing RT, the hands of the case were not fully protected during the manual injection of radioactive isotopes into the radioactive source, thus exposing him to radiation. This radiation exposure occurred consistently during the 3-day period in which a patient with cervical cancer was hospitalized for wound dressing and daily examination, and during the radioactive source removal. The case was also responsible for insertion and removal of radioactive isotopes in patients with esophageal or tongue cancer.’

### Exposure level assessment

**Fig. 2:**
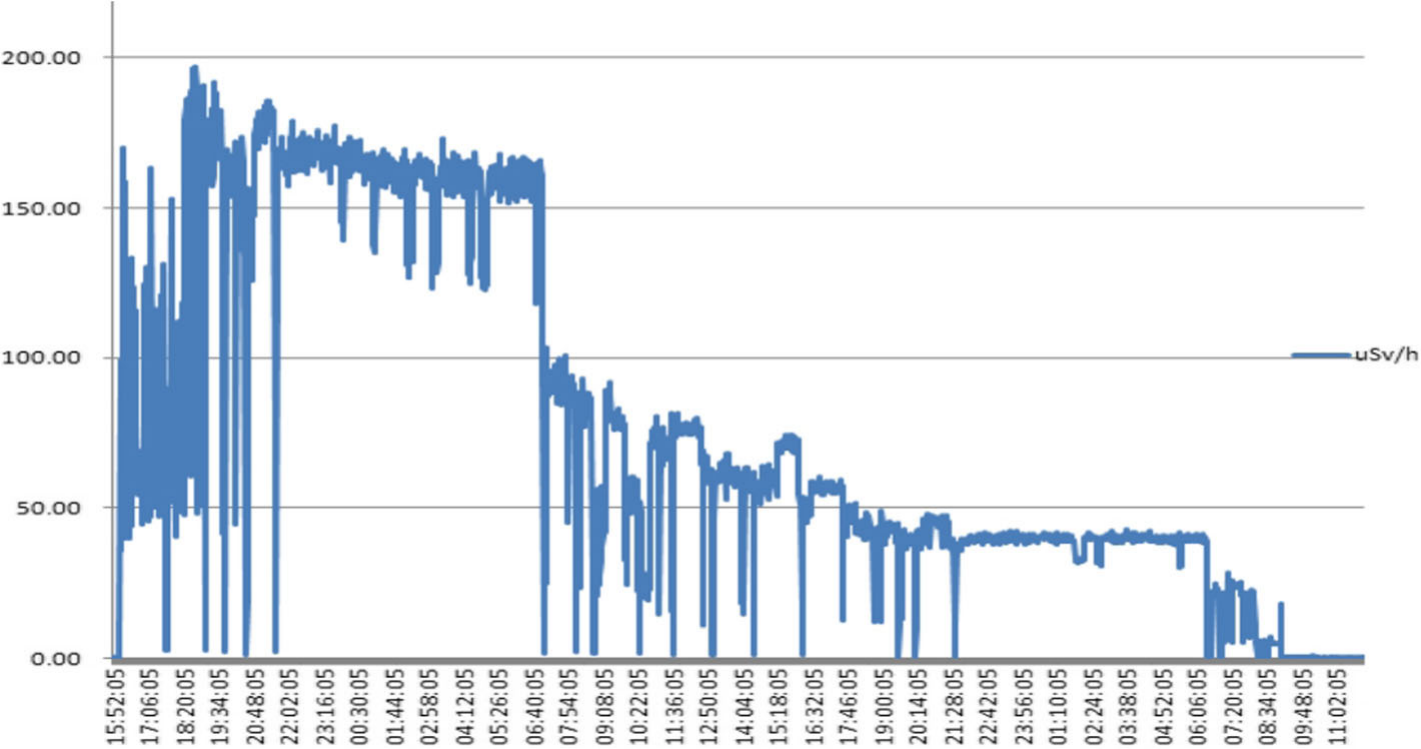
Radiation exposure assessment during radioiodine treatment of the thyroid: Radiation exposure assessment data provided by the National Hospital

The case would visit and observe the process of radioactive source production (tandem, ovoid) at a lab in the department of radiation oncology. He would then go to the hospital room in which he performed the interventions. A dome-shaped shielding wall was placed on a patient’s bed. It took approximately one minute per patient to manually open the shielding wall and prepare for the treatment. It took an additional minute to receive the radioactive source from the staff in charge, insert it, and pack the wound created by the insertion with gauze. The total duration of radiation exposure during the treatment was less than 3 min. The radioactive source was removed one day after the treatment. The removal took just as long as the insertion. This process of inserting and removing a radioactive source is no longer performed manually, but has been automated to prevent clinician’s exposure to radiation. The case was in full charge of this process during his work period, and performed treatment mainly in hospital room numbers 633 and 733. Table 1 shows the radiation exposure levels in one of the hospital rooms where the case performed treatments in 2001 including radiation doses measured at 1 m and 0.3 m above a patient’s bed. The tool of measurement was Eberline portable ESP-2 GM counter with Eberline HP-270 radiation detecter GM meter. The individual cumulative exposure doses of senior coworkers, two specialists including the case, and the person in charge of measuring radiation doses are listed. The cumulative exposure dose of the case was 10.29 mSv. The exposure dose was highest at in 2001 while he was working at the A National Hospital (2.22 mSv), and was higher than that of his coworkers in the same department. Comparison with coworkers showed that the case’s exposure dose was similar to that of those in charge of producing radioactive sources (10.55 mSv) and of senior coworkers with five more years of work experience than the case (12.21 mSv). Because humans no longer perform radioactive source insertion, we could not measure the level of radiation exposure in current workplaces. However, when we measured the level of radiation exposure during radioiodine therapy of the thyroid in which radioactive isotopes are still manually injected, we could see a peak in the radiation dose immediately after the injection of radioactive isotopes, which was followed by a gradual decrease, as shown in Fig. 2. Even if an individual’s cumulative radiation dose is lower than the exposure standard, the exposure dose can become relatively and significantly high immediately after the individual handles radioactive isotopes.

**Table 1:** Spatial dose measurement record in the Cs-137 treatment room [Unit: mSv]

Date	Room no.	Measuring point			Room no.	Measuring point			Room no.	Measuring point		
		Entrance	1 m	0.3 m		Entrance	1 m	0.3 m		Entrance	1 m	0.3 m
Jan.10.2001	533	0.04	0.25	1.60	633	0.04	0.25	2.15	733	0.04	0.25	2.20
Feb.09.2001	533	0.04	0.15	1.15	633	0.04	0.25	2.10	733	0.04	0.20	2.10
Feb.16.2001	533	0.04	0.20	1.35	633	0.04	0.32	2.40	733	0.04	0.25	2.30
Mar.09.2001	533	0.04	0.15	1.40	633	0.04	0.40	2.55	733	0.04	0.30	2.45
Mar.23.2001	533	0.04	0.10	1.20	633	0.04	0.20	2.10	733	0.04	0.32	2.45
Apr.13.2001	533	0.04	0.15	1.45	633	0.04	0.25	2.15	733	0.04	0.25	2.28
Apr.27.2001	533	0.04	0.20	1.30	633	0.04	0.30	2.20	733	0.04	0.32	2.40
May.10.2001	533	0.04	0.20	1.25	633	0.04	0.20	2.10	733	0.04	0.20	2.30
May.24.2001	533	0.04	0.25	1.35	633	0.04	0.30	2.30	733	0.04	0.25	2.25
Jun.07.2001	533	0.04	0.25	1.40	633	0.04	0.25	2.25	733	0.04	0.25	2.30
Jun.26.2001	533	0.04	0.10	1.25	633	0.04	0.25	2.30	733	0.04	0.30	2.45
Jul.12.2001	533	0.04	0.25	1.20	633	0.04	0.35	2.30	733	0.04	0.30	2.40
Jul.26.2001	533	0.04	0010	1.30	633	0.04	0.35	2.45	733	0.04	0.32	2.40
Aug.23.2001	533	0.04	0.20	1.25	633	0.04	0.20	2.10	733	0.04	0.25	2.30
Sep.06.2001	533	0.04	0.25	1.30	633	0.04	0.25	2.45	733	0.04	0.30	2.45
Sep.20.2001	533	0.04	0.20	1.25	633	0.04	0.32	2.30	733	0.04	0.25	2.30
Oct.11.2001	533	0.04	0.25	1.30	633	0.04	0.25	2.30	733	0.04	0.30	2.40
Oct.18.2001	533	0.04	0.15	1.45	633	0.04	0.30	2.10	733	0.04	0.25	2.30
Nov.08.2001	533	0.04	0.20	1.30	633	0.04	0.32	2.55	733	0.04	0.30	2.30
Nov.29.2001	533	0.04	0.22	1.50	633	0.04	0.32	2.20	733	0.04	0.35	2.45
Dec.06.2001	533	0.04	0.15	1.20	633	0.04	0.35	2.60	733	0.04	0.32	2.30

Radiation doses measured at 3 point: the entrance, 0.3 m and 1 m above bed, respectively, in hospital room number 533, 633, and 733. The case performed treatment in room number 633 and 733

## Discussion

Lymphocyte malignancies encompass a broad range of diseases, from cancers with good prognoses to rapidly advancing disease. Lymphocyte malignancies occur because of problems in the differentiation of immune cells and are associated with various clinical and histological findings. They are common among children aged 15 years or younger and only 20% of lymphocyte malignancies occur in adults. Although acute lymphoblastic leukemia has a complete recovery rate of 80% among children, it has a high relapse rate among adults, possibly because acute lymphoblastic leukemia is frequently accompanied by antibiotic resistance and chromosomal abnormalities with poor prognoses in adults. The Philadelphia translocation occurs frequently in adults with acute lymphoblastic leukemia and is closely associated with prognosis. The Philadelphia chromosome is a genetic abnormality in which the *ABL* gene on chromosome 9 fuses with the *BCR* gene on chromosome 22, which leads to an abnormal activation of tyrosine kinases and, in the long-term, leukemia. It was first discovered in a patient with chronic myelocytic leukemia and was later discovered in patients with acute lymphoblastic leukemia after 1970. It is found in approximately 20% of adults and is associated with an extremely poor prognosis in most patients [5].

The clinical symptoms of acute lymphoblastic leukemia appear suddenly because of inhibition of the proliferation of red blood cells, white blood cells, and platelets. Major symptoms resemble those of anemia such as dizziness, headaches, fatigue, infection, and fever, and bleeding symptoms such as epistaxis and bleeding gums. In some patients, leukemic cells infiltrate major organs, such as the liver, spleen, lymph nodes, central nervous system, bones, and lungs, and induce organ dysfunction or pain. When abnormal levels of blood cells are measured in a peripheral blood test and are accompanied by the aforementioned symptoms, a bone marrow test must be performed to obtain a definite diagnosis. Once a patient is diagnosed with acute lymphoblastic leukemia, supplementation of deficient blood components and chemotherapy must be performed as early as possible. Patients who do not undergo this treatment often die within 3 months after diagnosis due to bacterial infections or bleeding [5].

There are a few explanations as to how radiation causes leukemia, but the most widely accepted is that ionizing events occur within the body following irradiation. During this process, water is broken down to produce free oxygen, which induces DNA damage and results in temporary or permanent changes in irradiated cells. DNA that is damaged from irradiation, if not degraded, can give rise to mutations, and continued proliferation of mutant cells can lead to cancer cell development [6].

The cumulative exposure dose of the case could be estimated by using records of personal exposure doses, spatial doses measured at the case’s workplace during his work period, and radiation doses. In this case, the lifetime cumulative dose found through personal exposure dose records was 10 mSv, and the cumulative dose during the period in which the case worked at the A National Hospital was 2.92 mSv. The spatial dose measured at 0.3 m above a patient’s affected area, or his/her bed, during the work period was 2.5 mR/h on average. The bedside treatment of a patient lasted approximately 5–10 min. The case treated 25 patients per month on average during the same period. By assuming that each patient underwent irradiation at least twice, the exposure dose can be estimated to be 2.5 mR/h × 5–10 min × 2 × 25 patients/month = 625–1250 mR/h. An exposure dose of 1 μR/h is equivalent to an absorbed dose of 8.77 nGy/h in the air, which can be converted to an effective dose of 9.73 nSv/h. Therefore, the effective dose for the case is approximately 6.08–12.16 mSv.

However, such an estimation of effective dose has many limitations. Firstly, since the calculation of effective dose was calculated using only the number of patients who were discharged instead of treated, we cannot determine how many patients were actually treated. In addition, because we could not find the exact number of times that the case was exposed to radiation, we had to assume that he had been exposed to radiation at least twice per treatment, considering that each treatment involved insertion and removal of the radioactive source. The actual exposure frequency might be higher if we consider the fact that the case was also responsible for applying dressings to his patients during the period from the insertion of radioactive substances to their removal. Furthermore, we did not consider the radiation exposure that occurred while preparing for treatment and after opening the sealed radioactive source, and the distance between the clinician and his patients. Although we assumed that the treatment duration was 5–10 min per patient, the duration might vary based on the case’s level of skill and experience and each of his patient’s anatomical characteristics. Lastly, to protect patient privacy, the spatial dose measurements were not made during RT, and therefore may not be accurate. Although the exact radiation dose used for the case could not be found, we predict that it is larger than the estimated value that was calculated based on the minimum exposure.

A previous study by Savage on the correlation between acute lymphoblastic leukemia and radiation exposure reported chromosomal changes at low radiation doses below the recommended dose of 50 mSv and a linear relationship between radiation dose and chromosome abnormalities [7]. Evans et al. conducted a study on 197 workers exposed to radiation at less than 50 mSv/year over 10 years. They reported that chromosomal changes were linearly correlated with radiation dose and had no correlation with age. After comparing workers with radiation exposure and healthy adults, Balasem reported significant chromosomal changes even after a radiation exposure of less than 50 mSv [8]. Wilkinson and Dreyer performed a multivariate analysis of research data related to nuclear power plant workers and reported that the risk of leukemia in workers who were exposed to 10-50 mSv of radiation was 1.5 times that of workers who were exposed to less than 10 mSv of radiation [9]. In a cohort study of 8318 laboratory staff members who handled radioactive isotopes or radiation for energy research and development, the total cumulative exposure dose was found to be 17.3 mSv, below the acceptable level of exposure. However, the risk of leukemia in radiation workers who monitored exposure doses was 2.2 times higher than that of the general population (standardized mortality ratio = 2.23) [10]. A cohort study of 95,673 nuclear plant workers from the United States, United Kingdom, and Canada was conducted by the International Agency for Research on Cancer. The excess relative risk (ERR) and relative risk (RR) of leukemia excluding chronic lymphocytic leukemia (CLL) were 2.18 (90% confidence interval [CI] 0.1–5.7) and 1.22 (90% CI 0.1–5.7), respectively, both of which were significantly high [11]. The same researchers conducted a cohort study on 407,391 nuclear power plant workers from 15 countries. The ERR of leukemia excluding CLL was 1.93 per Sv in an analysis of 5.2 million man-years [12]. A cohort study by Kithhara of 22,039 medical radiation technologists, who performed radiation therapies including brachytherapy and radioisotope therapy between 1994 and 1998, reported that the RRs of epidermoid carcinoma and myocardial infarction among radiation technologists who performed brachytherapy were 1.29 and 1.37, respectively. The RRs of all other causes of mortality and cancer were 1.10 and 1.20, respectively [13]. Liu and Yoshinaga reported higher incidence and mortality rates of leukemia among health care providers with occupational exposure to radiation [14]. Similarly, several other studies reported significantly high risk of skin cancer, lung cancer, pancreatic cancer, and leukemia among doctors registered in the Society of Radiographers in the United Kingdom [15–20].

### Assessment of work-relatedness

The risk of lymphohematopoietic cancers excluding CLL has been reported to increase due to radiation exposure. The latent period from the initial irradiation to the onset of cancer can vary in length. In general, the risk of cancer is highest 5–10 years after irradiation and returns to normal pre-irradiations levels 25 years after irradiation [21]. Although no safe range of exposure dose has been identified, many studies claim that a single radiation dose of 50 rads or greater is carcinogenic. Although it remains controversial whether the risk of leukemia increases by exposure to radiation of less than 50 mSv, it has been reported that chromosomal changes occur at doses of radiation lower than the yearly recommended dose and have a linear relationship with the radiation exposure dose [21].

The case’s personal exposure dose during his time at the A National Hospital as a radiologic oncologist was estimated at 6.08–12.16 mSv based on the number of patients discharged per month, number of treatments performed per patient, treatment duration, and spatial exposure measurement records.

To assess the degree to which cancer was caused by radiation exposure among workers who developed cancer, probability of causation (PC) was calculated. PC greater than 50% indicated that radiation exposure was a cause of cancer. The following equation was used to determine PC.

$$ \mathrm{Probability}\ \mathrm{of}\ \mathrm{causation}=\mathrm{ERR}/\left(1+\mathrm{ERR}\right)\%100 $$

ERR is the excessive relative risk, which signifies the ratio of the risk of cancer increased as a result of radiation exposure to the baseline cancer risk in a non-exposed group. ERR can be estimated based on cancer patients’ gender, age at exposure, radiation exposure dose, elapsed time since exposure, and level of smoking. However, the 95% upper confidence limit of the probability of causation (PC) in this case was found to be 7.57%, and the probability of cancer developing due to radiation exposure was lower than the currently accepted probability of 50%.

There is another method to assess effect of radiation on cancer development. Jung et al. proposed a method to assess the work-relatedness of cancer in workers engaged in radiation work [22]. This method uses the screen dose of reliability a%. The screen dose of a% is the dose at which the upper confidence level of the probability of causation, a%, reaches 50% of the baseline probability of causation of radiation. When the exposure dose of a cancer patient is less than the screen dose, the patient’s demand for compensation can be dismissed. When the exposure dose exceeds the screen dose, the probability of causation is thoroughly calculated to determine the amount of compensation [23]. Jang et al. reported the 90%, 95%, and 99% screen dose for leukemia (not including chronic lymphocytic leukemia) to be 20.33 mSv, 17.38 mSv, and 12.93 mSv, respectively. The cumulative exposure dose of the patient in this study between 1996 and 2012 was 10.29 mSv, and the case’s highest yearly exposure dose during the period of employment at the A National Hospital was 2.22 mSv. This dose was lower than the screen dose, meaning that the probability of causation was less than 50% in this case [22].

However, since the space in which the patient performed treatment in the past has undergone renovation and treatment procedures have become automated, this method of radiation exposure estimation is limited in its accuracy. Considering the fact that the personal exposure dose was estimated based on the minimum exposure and a few others, it is highly likely that the actual personal exposure dose is higher than the estimated value. Firstly, the case’s hands were exposed to radiation during the process of manually injecting radioactive isotope (Cs-137). However, no records of receiving treatment for skin lesions including skin discoloration on the hands were found from the review of medical records of this case. In addition, it was impossible to ask the patient whether he had skin lesions or discoloration during the period of his employment through an interview since the case had already passed away. As such, there was no way to investigate whether the patient developed mild skin lesions during the period of his employment. However, based on the fact that the patient experienced weight loss, fatigue, and loss of appetite in November 2000, during which period the case worked at the A National Hospital, and that he could not wear any protective equipment on the hands such as lead gloves while he handled radioactive isotopes, it can be deduced that the case was exposed to radiation through his hands. This also explains why the case’s lifetime cumulative dose was close to that of technicians who produce radioactive sources and higher than that of his coworkers.

In addition, owing to the nationwide doctors’ strike at the time, doctors in the department of radiation oncology in the A National Hospital who did not go on strike were suddenly in charge of a very large number of patients. As one of these doctors, the case had to perform brachytherapy for 150 patients (estimated) in 6 months, and he worked 7 days per week, 10 h per day, without requesting any substitute doctors. This might have translated into the highest lifetime cumulative dose of 2.22 mSv during the patient’s work period.

Moreover, considering the increased time of radiation exposure due to increased workloads resulting from the doctors’ strike, the possibility that the case did not wear protective equipment, and the possibility that the case lost or did not wear the personal radiation dosimeter, the cumulative exposure dose could be higher than 2.22 mSv. An annual report of radiation exposure doses among workers engaged in radiation work released in 2015 reports that the number of workers who lost their personal radiation dosimeters and received new ones increased from 992 in 2014, to 1239 in 2015, marking a 24.9% increase relative to the previous year, and shows that losing a dosimeter is not an uncommon phenomenon among workers engaged in radiation work [2].

Finally, considering that the lead shielding gowns that clinicians wear during RT are only 60–80% effective [24], the actual radiation to which the case was exposed might be higher than the estimated dose.

## Conclusions

Although the cumulative exposure dose was lower than the radiation dose standard, the possibility of excessive radiation exposure over a short period of time, rather than the cumulative exposure level, should be considered in this case, because the level of radiation exposure was highest immediately after the case handled radioactive substances. In addition, the period between the occupational exposure to radiation and the onset of cancer closely matched the latent period of acute lymphoblastic leukemia of 5–10 years, suggesting that acute lymphoblastic leukemia has a probable of work-relatedness. Additional risk assessment of radiation exposure among clinicians in the future is therefore recommended.

## Abbreviations

CI: Confidence interval
CLL: Chronic lymphocytic leukemia
ERR: Excess relative risk
RR: Relative risk
RT: Radiation therapy
